# Pharmacokinetic/pharmacodynamic assessment of a novel, pharmaceutical lipid–aspirin complex: results of a randomized, crossover, bioequivalence study

**DOI:** 10.1007/s11239-019-01933-7

**Published:** 2019-08-16

**Authors:** Dominick J. Angiolillo, Deepak L. Bhatt, Frank Lanza, Byron Cryer, Jin-fei Dong, Walter Jeske, Ronald R. Zimmerman, Estela von Chong, Jayne Prats, Efthymios N. Deliargyris, Upendra Marathi

**Affiliations:** 1 Division of Cardiology, University of Florida College of Medicine, 655 West 8th street, Jacksonville, FL 32209 USA; 2 Brigham and Women’s Hospital Heart & Vascular Center, Harvard Medical School, Boston, MA USA; 3 Houston Institute for Clinical Research, Houston, TX USA; 4 University of Texas Southwestern Medical School, Dallas, TX USA; 5 Division of Hematology, Department of Medicine, University of Washington, Member, BloodWorks NW Research Institute, Seattle, WA USA; 6 Cardiovascular Research Institute, Loyola University Chicago Health Sciences Division, Maywood, IL USA; 7 PLx Pharma, Sparta, NJ USA; 8 Elysis LLC, Carlisle, MA USA; 9 7 Hills Pharma, Houston, TX USA

**Keywords:** Aspirin, Pharmacodynamic, Pharmacokinetic, Platelet, Bioequivalence

## Abstract

Aspirin (acetylsalicylic acid, ASA) can lead to gastrointestinal mucosal injury through disruption of its protective phospholipid bilayer. A liquid formulation of a novel pharmaceutical lipid–aspirin complex (PL-ASA) was designed to prevent this disruption. We sought to determine the pharmacokinetic (PK)/pharmacodynamic (PD) characteristics of PL-ASA compared with immediate release aspirin (IR-ASA). In this active-control crossover study, 32 healthy volunteers were randomized to receive 1 of 2 dose levels (a single dose of 325 mg or 650 mg) of either PL-ASA or IR-ASA. After a 2-week washout period between treatment assignments, subjects received a single dose of the alternative treatment, at the same dose level. The primary objectives of the study were to assess, for PL-ASA and IR-ASA at 325 mg and 650 mg dose levels, PK and PD bioequivalence, and safety, over a 24–h period after administration of both drugs. PK parameters were similar for PL-ASA and IR-ASA, and met FDA-criteria for bioequivalence. Regarding PD, both drugs also showed C_min_ TxB2 values below 3.1 ng/mL (cut-off associated with decreased cardiovascular events) and > 99% inhibition of serum TxB2 ( ≥ 95% inhibition represents the cut-off for aspirin responders) along with similar results in several secondary PK/PD parameters. There were no serious adverse events or changes from baseline in vital signs or laboratory values in either of the 2 treatment groups. PL-ASA’s novel liquid formulation has similar PK and PD performance compared with IR-ASA, supporting functional and clinical equivalence. These data coupled with the improved gastric safety of PL-ASA suggest that this novel formulation may exhibit an improved benefit-risk profile, warranting evaluation in future trials.

*Clinical trial registration:*http://www.clinicaltrials.gov. Unique Identifier: NCT04008979

**Electronic supplementary material:**

The online version of this article (10.1007/s11239-019-01933-7) contains supplementary material, which is available to authorized users.

## Highlights

- Observational studies of upper gastrointestinal (GI) bleeding in patients taking aspirin showed that the risk is not lowered with coated tablets; PD studies also suggest high rates of non-responsiveness.
- The novel pharmaceutical lipid-aspirin-complex (PL-ASA) liquid capsule formulation has been specifically designed to reduce the acute GI toxicity of aspirin.
- The results of the current study demonstrate functional and clinical bioequivalence between immediate release aspirin and PL-ASA and suggest that this novel aspirin formulation with significantly lower risk of acute GI toxicity can also deliver aspirin in a fast and reliable manner that results in the desired antiplatelet effect.
- Further studies are warranted to test the performance of PL-ASA versus enteric-coated aspirin, the preferred aspirin by the majority of physicians and patients.

## Introduction

Aspirin, or acetylsalicylic acid, is an irreversible inhibitor of the platelet cyclooxygenase (COX)-1 enzyme, which leads to suppression of thromboxane (Tx) A2 formation, a potent inducer of platelet activation and aggregation [1]. Low-dose aspirin regimens (75 to 325 mg) are commonly used for the prevention of serious vascular events in patients with established atherosclerotic disease [2].

Although recent data have questioned its role for primary prevention, low-dose aspirin still remains a cornerstone of guideline-recommended therapy for secondary prevention [2–5]. High-dose aspirin ( ≥ 650 mg) has antipyretic, analgesic and anti-inflammatory properties, which are primarily mediated through inhibition of COX-2 by salicylic acid, aspirin’s primary metabolite [1]. In addition to the established efficacy, aspirin is also frequently associated with gastrointestinal (GI) toxicity and bleeding, which can lead to poor adherence or discontinuation and increase the risk of ischemic recurrences [6–8].

Aspirin-induced GI toxicity is mediated by three mechanisms including: (a) impaired hemostatic function via antiplatelet effects (b) inhibition of COX-derived prostaglandin (PG) production (locally and through systemic exposure), which is key in epithelial mucus production, microvascular mucosal perfusion and wound healing in the GI tract, and (c) physical disruption of the protective gastric phospholipid barrier thereby allowing direct acid injury [9]. With an estimated 40 million Americans consuming daily aspirin, GI-related toxicity associated with aspirin use represents an important public health problem [10]. These observations underscore the need for aspirin formulations with a more favorable safety profile while maintaining pharmacologic efficacy.

PL-ASA is a novel pharmaceutical lipid–aspirin complex liquid formulation developed to mitigate disruption of the epithelial phospholipid layer of the gastric mucosa without delaying absorption [11–13]. This study was a prospective pharmacokinetic (PK) and pharmacodynamic (PD) evaluation conducted to define bioequivalence and support the new drug application and subsequent approval of PL-ASA by the United States Food and Drug Administration (FDA). To determine bioequivalence for FDA approval, the study was performed with healthy volunteers, and the doses studied bracketed currently approved doses for aspirin for cardiovascular protection (i.e., 325 mg) and anti-inflammatory effects (i.e., 650 mg).

## Methods

### Study design and study population

This current investigation was a randomized, active control, cross-over study to assess the bioequivalence (using PK and PD parameters), and safety of PL-ASA compared with IR-ASA administered orally at a single dose (Clinicaltrials.gov identifier: NCT04008979). The study design and details of the study conduct are provided in the Supplemental Appendix (Online Fig. 1, Online Table 1, and “Statistical methods”). A total of 32 healthy subjects were randomized to treatment with 1 of 2 dose levels (325 mg or 650 mg) of either IR-ASA tablets (Genuine Bayer® Aspirin) or PL-ASA capsules. After a 2-week washout period between treatment assignments, subjects received a single dose of the alternative treatment, at the same dose level. Therefore, in aggregate, each subject received a total of two doses of study drug (one dose of PL-ASA and one dose of IR-ASA at the same dose level) separated by a 2-week washout period between the doses.

All subjects were included in the safety analyses, which included monitoring incidence of adverse events, and changes in laboratory assessments (hematology and blood chemistry) and vital signs. The study protocol was approved by IntegReview Ethical Review Board (IORG Number IORG0000689) and conducted in the Houston Institute for Clinical Research. Dr Angiolillo prepared the first draft of this manuscript, and had full access to the analyzed data.

### PK and PD analyses

After absorption, as acetylsalicylic acid is rapidly converted to salicylic acid by hydrolysis and first-pass metabolism, peak plasma concentrations of acetylsalicylic acid are extremely sensitive to minor variations in solid dosage form dissolution and disintegration. In contrast, plasma concentrations of salicylic acid are predictable and relatively stable, and salicylic acid is considered to be a superior analyte for comparative bioequivalence assessments. Salicylic acid PK parameters were used for primary PK endpoints.

The primary PK endpoints were the parameters AUC_0-t_, AUC_0-∞_, C_max_, t_max_, λ_z_, t_½_, V_D_/F, and CL/F of the primary PL-ASA/IR-ASA metabolite salicylic acid, and the primary PD endpoints were AUC_0-24_, I_max_, and t_max_ of the percent inhibition of serum Thromboxane B2 (TxB2) levels. PK and PD parameters were used to assess bioequivalence [14]. Secondary PK endpoints included similar PK parameters for acetylsalicylic acid; secondary PD endpoints assessed the incidence of aspirin responders and the level of platelet aggregation in response to arachidonic acid and collagen in an ex vivo assay. The full list of PK and PD endpoints measured are given in Online Table 1.

### Statistical methods

PK analyses were performed on all 32 subjects. For the comparisons between PL-ASA and IR-ASA, confidence intervals (CI) of 95% were employed, and statistical significance was assessed using a two-sided test at the 0.05 significance level. For bioequivalence analyses, 90% CI were employed, and statistical significance was assessed using 2 one-sided tests at the 0.05 significance level. Further details of the statistical analysis methods are given in the Supplemental Appendix.

### Analytical methods

Plasma samples were collected into sodium fluoride/potassium oxalate, frozen (-80 °C) and analyzed by MEDTOX Laboratories, St. Paul, MN. Salicylic acid and acetylsalicylic acid plasma levels and TxB2 serum levels were determined by High Performance Liquid Chromatography with tandem Mass Spectrometry (LC- MS/MS). Urinary 11-dehydro-TxB2 was determined by an Enzyme-Linked Immunosorbent Assay (ELISA), and urinary creatinine was measured via a spectrophotometric assay. Subjects were considered as being aspirin responders if: (a) demonstrated ≥ 95% inhibition of serum TxB2 levels (b) had TxB2 C_min_ values < 3.1 ng/mL, or c) had values of urinary 11-dehydro-TxB2 ≤ 1500 pg/mg of creatinine [15–17]. Inhibition of platelet aggregation in response to arachidonic acid (0.5 mg/mL) and collagen (4 µg/mL, fibrillar Type I) as agonists in blood samples collected after study treatment was measured by ex vivo light transmittance aggregometry.

## Results

### Pharmacokinetic results

**Fig. 1:**
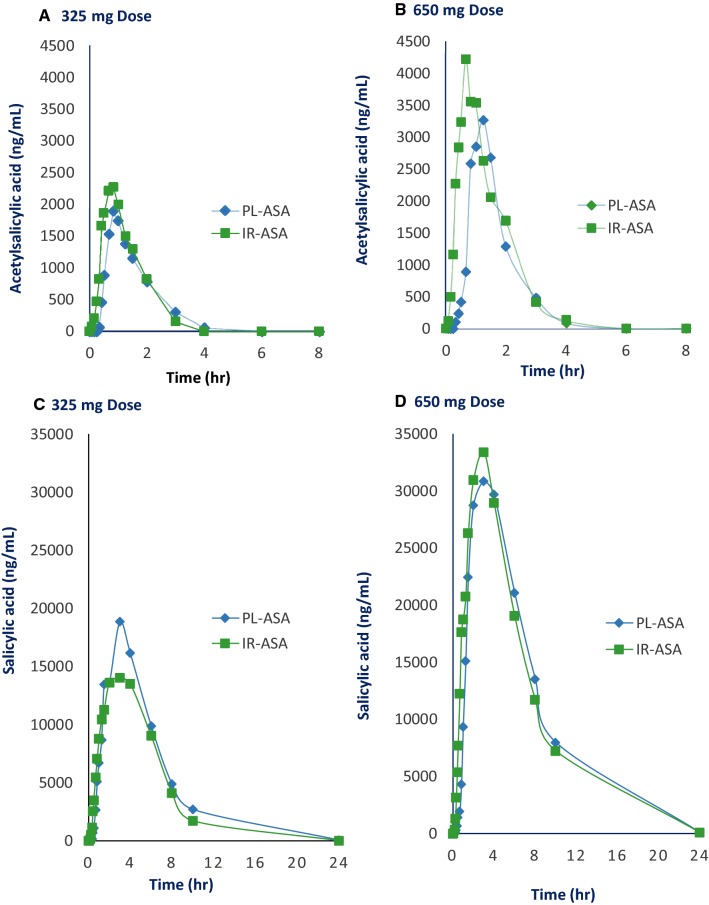
Impact of the pharmaceutical formulation on aspirin’s disposition. Plasma concentration time profiles of acetylsalicylic acid (**a**, **b**) and salicylic acid (**c**, **d**) were compared in healthy, fasted volunteers who received single doses (325 and 650 mg) of PL-ASA and IR-ASA. *h* hours, *IR-ASA* immediate release aspirin, *mg* milligrams, *ng* nanograms, *PL-ASA* pharmaceutical lipid–aspirin complex

The bioavailability of aspirin was estimated using the stable active metabolite plasma salicylic acid. Concentration–time curves for acetylsalicylic acid and salicylic acid levels illustrate that time to mean peak acetylsalicylic acid concentration after PL-ASA appears to be nominally longer and mean peak acetylsalicylic acid concentration slightly lower compared with IR-ASA, however, none of these differences were statistically significant and both formulations were absorbed and metabolized at similar rates (Fig. 1).

For the primary endpoints (the salicylic acid PK parameters AUC_0−t_, AUC_0–∞_, C_max_, t_max_, λ_z_, t_½_, V_D_/F, and CL/F), the PK parameter values were similar for PL-ASA and IR-ASA, but the median AUC_0−t_ and C_max_ values were nominally slightly higher for the PL-ASA group than for the IR-ASA group at the 325 mg dose level (Table 1); at the 650 mg dose level, the median AUC_0−t_, AUC_0–∞_, and C_max_ values are slightly higher following IR-ASA treatment (Table 1). Comparable results were obtained by analysis of PK parameters based on acetylsalicylic acid, a secondary endpoint: the acetylsalicylic acid PK parameters for both study drugs were similar, but the median AUC_0−t_ and AUC_0–∞_ values were nominally slightly higher for the IR-ASA group and median C_max_ values are nominally slightly higher following PL-ASA at both dose levels (Online Table 3).

**Table 1:** Pharmacokinetic salicylic acid parameters after administration of PL-ASA and IR-ASA in healthy volunteers

Parameter	PL-ASA (n = 13)		IR-ASA (n = 13)	
	Median	Range	Median	Range
325-mg dose^a^				
AUC_0−t_ (μg × min/mL)	5489	2968–10,795	5401	2736–13,855
AUC_0–infinity_ (µg × min/mL)	5501	3123–13,239	5801	2863–13,964
C_max_ (μg /mL)	19	10–27	16	10–25
t_max_ (min)	120	75–240	120	75–240
λ (1/min)	0.005	0.003–0.007	0.005	0.002–0.007
t _½_ (min)	143	98–249	151	99–353
650-mg dose				
AUC_0-t_ (μg × min/mL)	14,934	8248–23,332	15,444	7673–22,429
AUC_0–infinity_ (µg × min/mL)	14,856	8847–23,515	15,477	8150–22,926
C_max_ (μg /mL)	35	25–53	36	24–44
t_max_ (min)	180	120–360	180	75–240
λ (1/min)	0.005	0.003–0.006	0.005	0.003–0.006
t_½_ (min)	136	117–250	150	119–270

*AUC*_*0*−*t*_ area-under-the-curve, *AUC*_*0*−*∞*_ AUC_0−t_ extrapolated to infinity, *C*_*max*_ maximum plasma concentration, *IR-ASA* immediate release aspirin, *μg* micrograms, *min* minutes, *mL* milliliters, *n* number, *PL-ASA* pharmaceutical lipid–aspirin complex, *t*_*max*_ time of peak drug concentration, *λ*_*z*_ terminal elimination rate constant, *t*_*½*_ first-order elimination half-life

^a^PK population does not include 2 subjects who did not receive all planned doses, and one subject whose dosing was not as protocol-specified

Values of the log-transformed PK parameters AUC_0−t_, AUC_0–∞,_ and C_max_ for the ratio of PL-ASA to IR-ASA were examined to evaluate bioequivalence of PL-ASA and IR-ASA at both dose levels. The results of these analyses are presented in Table 2. Analysis of the PK endpoints showed that the 90% CIs for the mean log-transformed salicylic acid parameters AUC_0−t_, AUC_0–∞_ and C_max_ for the ratio PL-ASA to IR-ASA at both the 325 mg and 650 mg dose levels were within the FDA-accepted bioequivalence range of 80% to 125%. All 90% CIs contained 100% at both dose levels, indicating that nearly the same amount of salicylic acid was metabolized by each subject.

Analysis of the acetylsalicylic acid PK parameters for bioequivalence demonstrated that the 90% CIs for the mean log-transformed acetylsalicylic acid ratio for AUC_0−t_ and AUC_0–∞_ were within the 80% to 125% range were within the 80% to 125% range at both dose levels (Table 2), but the 90% CI for C_max_ was outside the range at both dose levels.

A total of 32 subjects were randomized. All but two subjects, both in the 325 mg dose group, were crossed over to the second study drug. Three additional subjects, one in the 325 mg dose group and two in the 650 mg dose group, were excluded from PK assessments because of the inability to confirm assigned dosing in these subjects. Demographics of the study population are provided in Online Table 2. No serious adverse events (AE) were reported during the study; only 1 AE was reported ('flu syndrome'), in the PL-ASA group, graded as mild severity and considered be unrelated to the study drug. Vital signs and laboratory results were unremarkable.

**Table 2:** Bioequivalence parameters of PL-ASA and IR-ASA in healthy volunteers

	Ratio (%)^a^	90% CI^b^	P value^c^
Salicylic acid 325-mg dose (n = 13)			
AUC_0−t_ (μg × min/mL) (n = 13)	97	89–104	0.43
AUC_0-∞_ (µg × min/mL) (n = 13)	98	91–106	0.62
C_max_ (μg /mL) (n = 13)	104	92–117	0.59
Salicylic acid 650-mg dose			
AUC_0−t_ (μg × min/mL) (n = 14)	98	93–103	0.44
AUC_0-∞_ (µg × min/mL) (n = 14)	99	95–103	0.66
C_max_ (μg/mL) (n = 14)	106	97–115	0.25
Acetylsalicylic acid 325-mg dose			
AUC_0−t_ (μg × min/mL) (n = 13)	94	83–107	0.41
AUC_0-∞_ (µg × min/mL) (n = 7)	86	72–102	0.13
C_max_ (μg/mL) (n = 13)	114	81–159	0.51
Acetylsalicylic acid 650-mg dose			
AUC_0−t_ (μg × min/mL) (n = 14)	92	86–99	0.06
AUC_0-∞_ (µg × min/mL) (n = 11)	91	86–98	0.03
C_max_ (μg/mL) (n = 14)	105	76–145	0.78

Only subjects who received both treatments and whose appropriate dosing was verified are included

*AUC*_*0−t*_ area-under-the-curve, *AUC*_*0–∞*_ AUC_0−t_ extrapolated to infinity, *CI* confidence interval, *C*_*max*_ maximum plasma concentration, *IR-ASA* immediate release aspirin, *μg* micrograms, *min* minutes, *mL* milliliters, *n* number, *PL-ASA* pharmaceutical lipid–aspirin complex, *t*_*max*_ time of peak drug concentration, *λ*_*z*_ terminal elimination rate constant, *t*_*½*_ first-order elimination half-life

^a^Ratio = 100 × Geometric mean (PL-ASA)/geometric mean (IR- ASA)

^b^90% Confidence interval on the ratio of PL-ASA to IR-ASA

^c^ANOVA p-value for the difference in the treatment estimates

### Pharmacodynamic results

**Fig. 2:**
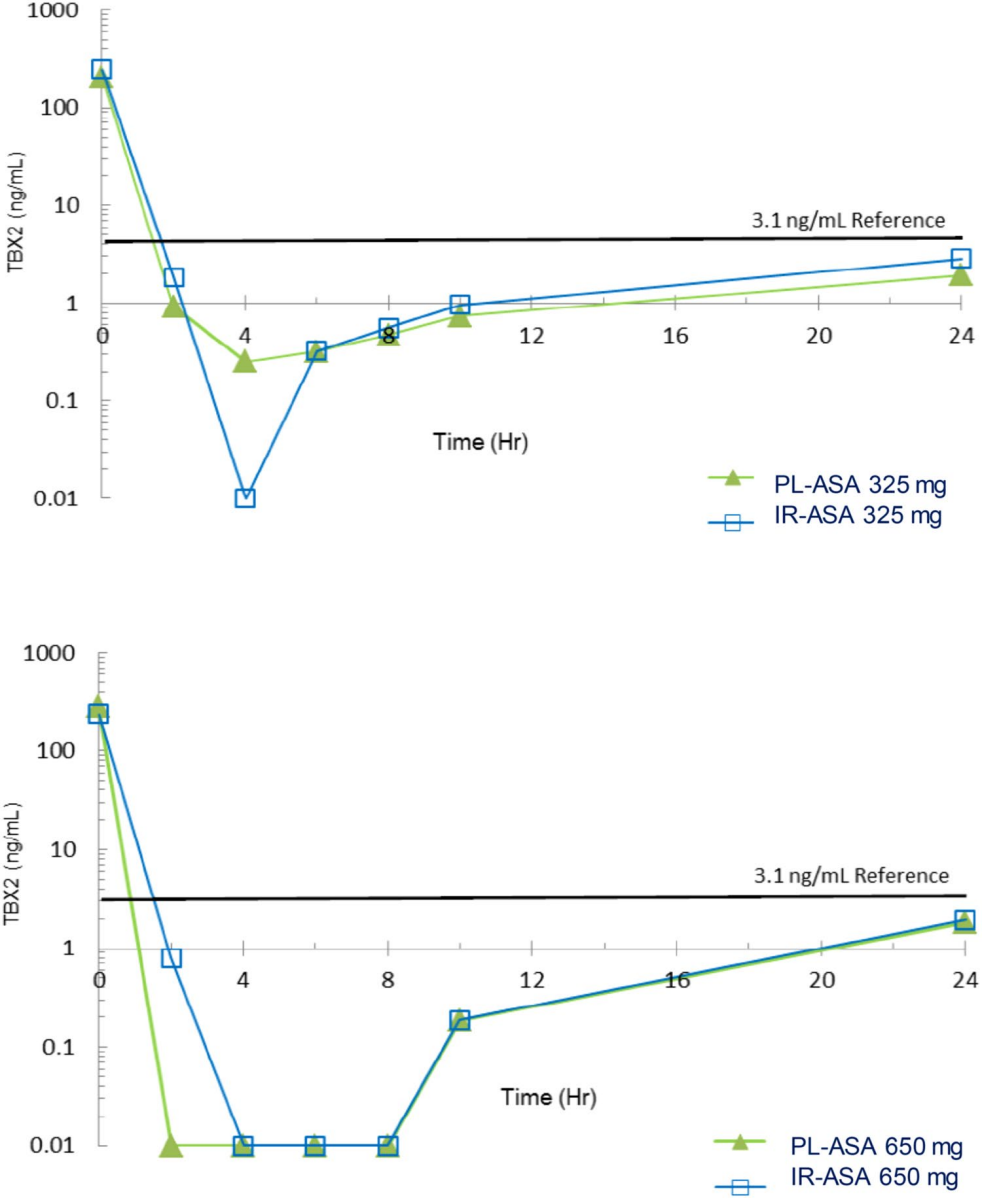
Graphs of the mean concentration of TxB2. Decrease in TxB2 level from baseline was measured as a marker for inhibition of platelet aggregation by PL-ASA and IR-ASA (samples collected at baseline, and 2, 4, 6, 8, 10, and 24 h post-dose). Results are shown as mean % inhibition of TxB2 concentration for 325 mg (top panel) and 650 mg dose (bottom panel). *h* hours, *IR-ASA* immediate release aspirin, *mg* milligrams, *mL* milliliters, *ng* nanograms, *PL-ASA* pharmaceutical lipid–aspirin complex, *TxB2* thromboxane B2

To establish pharmacodynamic equivalence between PL-ASA and IR-ASA we compared parameters related to COX-1 activity and platelet aggregation. Thromboxane B2 (TXB2), a downstream product and established surrogate for TXA2 production, was measured. The mean concentration over time following dosing with PL-ASA and IR-ASA were shown to be similar (Fig. 2). In addition, C_min_ (TxB2) values for both PL-ASA and IR-ASA were below 3.1 ng/mL (the cut-off associated with a decreased occurrence of major adverse cardiovascular events in patients taking aspirin for cardioprotection), suggesting that PL-ASA and IR-ASA could be considered functionally and clinically equivalent.

The PD parameters AUC_0-24_, I_max_, and t_max_ based on the percent inhibition of serum TxB2 levels for PL-ASA and IR-ASA were very similar, with identical I_max_ and t_max_ medians at both dose levels (Online Table 4). The 90% CIs for the mean log-transformed parameters AUC_0–24_ and I_max_ were within the accepted 80% to 125% bioequivalence interval at both dose levels. All the 90% CIs contained 100%, indicating little variation present in the percent inhibition of TxB2 levels observed among subjects. In addition, all subjects evaluable for this TxB2-related primary endpoint demonstrated at least 99% inhibition of baseline TxB2 levels, for both PL-ASA and IR-ASA at both dose levels, and were classified as aspirin responders (Online Table 5).

Levels of urinary 11-dehydro-TxB2, a stable metabolite of TxB2, also indicated a similar and high incidence of aspirin responders for both formulations (subjects having ≤ 1500 pg urinary 11-dehydro-TxB2 per mg of creatinine). Urinary 11-dehydro-TxB2 assay results showed that 85.7% of subjects were responders to IR-ASA and 78.6% were responders to PL-ASA at 325 mg doses; 100.0% responded to IR-ASA and 93.8% to PL-ASA at 650 mg doses.

**Fig. 3:**
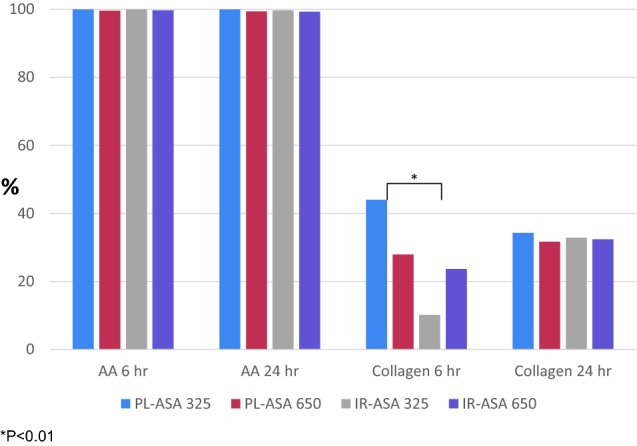
Mean Percent Inhibition of Platelet Aggregation (IR-ASA and PL-ASA doses of 325 mg and 650 mg). The baseline levels of platelet aggregation induced by arachidonic acid and by collagen in platelet-rich plasma (PRP) prepared from blood samples collected at screening were determined. Platelet aggregation induced by each agonist was then measured in PRP prepared from blood drawn at 6 h and at 24 h after dosing, and the % inhibition of aggregation observed was calculated for each subject at each time point (compared to baseline). *P < 0.01. *AA* arachidonic acid, *h* hours, *IR-ASA* immediate release aspirin, *PL-ASA* pharmaceutical lipid–aspirin complex

In platelet-rich plasma from subjects at baseline, and 6 and 24 h after PL-ASA or IR-ASA treatment at either dose level (Fig. 3), platelet aggregation induced by arachidonic acid was inhibited more than 99% compared to that observed in pre-treatment blood samples. When the same assay was used to measure collagen-induced platelet aggregation (a distinct process in the early stages of in vivo plaque-formation at the site of vascular damage), collagen-induced aggregation was inhibited to a lesser degree than arachidonic acid-induced aggregation. There were no statistically significant differences between the effects of PL-ASA and IR-ASA at the same dose and time point, except in the 6-h samples after dosing at 325 mg, where inhibition of platelet aggregation was greater with PL-ASA (44.0% vs. 10.2%, p < 0.01), a difference that was no longer present at 24 h (34.3% vs. 32.9, p = ns).

## Discussion

PL-ASA is a novel, pharmaceutical lipid-aspirin formulation administered in liquid-filled capsules, which was developed specifically to mitigate disruption of the epithelial phospholipid layer of the gastric mucosa and prevent direct gastric injury while still providing fast and complete absorption [13, 18]. The results of this PK/PD and bioequivalence study in healthy volunteers for PL-ASA compared with traditional IR-ASA can be summarized as follows:

(1) *PK* PL-ASA and IR-ASA have similar PK profiles, with the 90% CIs for the mean log-transformed salicylic acid PK parameters within the bioequivalence acceptance interval for both the 325 mg and the 650 mg dose levels. PK parameters for plasma acetylsalicylic acid levels were within the 80% to 125% bioequivalence range at the 325 mg dose level; at the 650 mg dose, only the 90% CIs for the mean log-transformed acetylsalicylic acid parameters AUC_0−t_ and AUC_0–∞_ were within the that range. Differences in PK parameters reflected a difference in the rate of absorption between PL-ASA and IR-ASA dosing, likely due to differences between the capsule/tablet disintegration rates of PL-ASA capsules and IR-ASA tablets. Because acetylsalicylic acid is rapidly converted to salicylic acid by hydrolysis and first-pass metabolism, peak plasma concentrations of acetylsalicylic acid are extremely sensitive to minor variations in solid dosage form dissolution and disintegration. In contrast, plasma concentrations of salicylic acid are predictable and relatively stable compared with acetylsalicylic acid, and thus salicylic acid is considered to be a superior analyte for comparative bioequivalence assessments. Overall, these PK findings support that PL-ASA is bioequivalent to IR-ASA.

(2) *PD* PL-ASA and IR-ASA have similar profiles at the 325 mg and 650 mg dose levels. All evaluable subjects for TxB2 levels experienced at least 99% inhibition by both drugs at both dose levels, clearly meeting the threshold of 95% inhibition of serum TxB2 suggested as an indication of complete aspirin responsiveness [15]. C_min_(TxB2) values for both PL-ASA and IR-ASA are below 3.1 ng/mL, a cut-off associated with a decreased occurrence of major adverse cardiovascular events in patients taking aspirin for cardiovascular protection [17]. PD equivalence was further supported by the observation of complete inhibition of arachidonic acid-induced platelet aggregation following both PL-ASA and IR-ASA administration at both dose levels.

Regarding inhibition of collagen-induced platelet aggregation, PL-ASA exhibited a higher inhibition rate than IR-ASA at 6 h post-dosing. Inhibition rates were similar for PL-ASA and IR-ASA at 24 h post-dosing. Our observations on the enhanced degree of collagen-induced aggregation with PL-ASA 325 mg at 6 h are likely play of chance given that the level of inhibition was even greater than with PL-ASA 650 mg and not observed in other investigations (data on file, PLXPharma). Thus, PL-ASA demonstrated overall anti-platelet activity equivalent to that of IR-ASA.

(3) *Safety* PL-ASA was shown to be safe; only 1 unrelated, mild AE was reported during the study; no serious AEs; and no clinically significant vital sign or laboratory abnormalities occurred.

Aspirin-induced GI toxicity is an important side effect associated with both short and long-term use of aspirin [19]. These observations have prompted investigations aimed at developing aspirin formulations with greater tolerability, such as enteric coated tablets [20]. However, enteric coating is known to delay and impair drug absorption and contribute to variability in PK/PD profiles [21–25]. PL-ASA is a novel formulation of aspirin, specifically developed to mitigate disruption of the epithelial phospholipid layer of the gastric mucosa without delaying absorption. PL-ASA has been shown to reduce acute gastric mucosal lesion formation during short-term exposure when compared with IR-ASA [18]. Moreover, PL-ASA is an immediate-release formulation. In fact, the present analyses of the plasma concentration profiles of aspirin and its metabolite salicylic acid confirms that PL-ASA behaves as an immediate release formulation in fasted healthy volunteers. We have previously demonstrated that the pharmacokinetics of PL-ASA are also similar to those of IR-ASA in an obese diabetic population [13]. Overall, a new aspirin formulation characterized by improved safety and comparable efficacy with IR-ASA can be very appealing for both the acute and chronic management of patients with vascular disease. In the acute setting, the favorable safety profile of PL-ASA and its prompt absorption are indeed characteristics that are highly desirable in patients presenting with acute coronary syndromes or ischemic stroke who will likely also be treated with additional antithrombotic agents, hence increasing risk of GI bleeding, but who also require immediate and effective antiplatelet therapy. In the chronic setting, reliable platelet inhibition coupled with better tolerability due to reduced GI toxicity can improve patient adherence and provide better long-term protection from recurrent events.

## Study limitations

The present bioequivalence study was conducted in healthy volunteers who did not have an indication to be treated with aspirin. This is consistent with standard guidance for FDA-required bioequivalence studies in order to allow for pure pharmacologic comparisons without the risk of interaction or interference by underlying clinical conditions. In addition, such bioequivalence studies are also not designed nor meant to test for clinical outcomes. However, the information derived from this study serves as a foundation to inform additional studies in specific patient populations that do have an indication to be treated with aspirin. It may be questioned why reporting of these early data supporting bioequivalence of PL-ASA with IR-ASA has occurred after publication of other reports on PL-ASA. Following FDA approval of PL-ASA, the prioritization shifted to studies focusing on antiplatelet activity (efficacy) and gastrointestinal toxicity (safety). However, in light of ongoing development of an 81 mg formulation and the upcoming commercial availability of both doses, investigators felt that publication of all prior data with PL-ASA and especially the data that supported FDA approval was very important.

## Conclusions

Administration of a single dose of PL-ASA at 325-mg or 650-mg is safe, and pharmacokinetically and pharmacodynamically equivalent to IR-ASA based on log-transformed ratios for AUC_0−t_, AUC_0–∞_, and C_max_ of plasma salicylic acid and for AUC_0-24_ and I_max_ of the percent inhibition of serum TxB2 levels. The findings of this study demonstrate that PL-ASA is both bioequivalent to aspirin for non-prescription indications, and pharmacodynamically equivalent to aspirin for physician-directed cardiovascular indications. The previously reported improved endoscopic safety profile of PL-ASA coupled with its pharmacologic efficacy equivalent to IR-ASA may potentially result in an improved benefit-risk profile. Further studies are warranted to test the performance of PL-ASA versus enteric-coated aspirin, the aspirin preferred by the majority of physicians and patients.

## Electronic supplementary material

Below is the link to the electronic supplementary material.

Supplementary file1 (DOCX 59 kb)
