# Supplementary material for: Pharmacokinetic/pharmacodynamic assessment of a novel, pharmaceutical lipid–aspirin complex: results of a randomized, crossover, bioequivalence study
Source: J Thromb Thrombolysis. 2019 Aug 16;48(4):554–62. doi: 10.1007/s11239-019-01933-7 (PMC6800884; doi:10.1007/s11239-019-01933-7)
Supplement: Supplementary file 1 — Supplementary file1 (DOCX 59 kb) [file 11239_2019_1933_MOESM1_ESM.docx]

**Supplemental Appendix**

**Pharmacokinetic/Pharmacodynamic Assessment of a Novel, Pharmaceutical Lipid-Aspirin Complex: Results of a Randomized, Crossover, Bioequivalence Study**

**Online Figure 1.** Study Design and Implementation

**Statistical Methods**

**Online Table 1** Primary and secondary PK and PD endpoints

**Online Table 2.** Demographics of the study population

**Online Table 3.** Pharmacokinetics of PL-ASA and IR-ASA (acetylsalicylic acid) in healthy volunteers

**Online Table 4.** Summary of Inhibition of TxB2 Parameters for PL-ASA and IR-ASA

**Online Table 5.** Summary of Log-Normalized Ratio of PL2200 to Aspirin for Statistics Based on Inhibition of TxB2 (325mg and 650mg doses)

**Online Table 6**. Incidence of Aspirin Responders as Assessed by % Inhibition of Serum Thromboxane B2 Levels

**Online Figure 1. Study Design and Implementation.**

PL-ASA=pharmaceutical lipid aspirin complex; IR-ASA= immediate release aspirin

The study protocol was approved by IntegReview Ethical Review Board (IORG Number IORG0000689) and conducted in the Houston Institute for Clinical Research from February 2008 to June 2008. .Study participants provided written informed consent. Healthy, non-smoking male and female volunteers (≥ 21 years) abstained from medications affecting platelet function for 2 weeks prior to beginning the study and throughout its duration. Subjects abstained from food for 10 hours prior to dosing (each dose given with 240 mL water), were provided a standard meal 4 hours after drug administration, and dinner was allowed 10 hours after administration.

After completion of the first treatment and a minimum of a 2-week washout period (range from 14 - 21 days), all but 2 subjects (in the 325mg dose group) were crossed over and received treatment with the alternative compound at the same dose level (i.e., subjects randomized to receive IR-ASA tablets as a first treatment received PL-ASA as the second treatment, and vice-versa).

Blood samples for evaluation of PK and PD variables were collected over a 24-hour period after drug administration. Laboratory assessments (hematology, blood chemistry) were performed at screening, just prior to each study drug administration (at Visits 1 and 3), and 24 hours after each study drug administration (at Visits 2 and 4). Adverse events and the use of concomitant medications were monitored throughout the study.

**Online Table 1. Primary and secondary PK and PD endpoints**

|  | **Endpoints** |
| --- | --- |
| **Primary** |  |
| salicylic acid  PL-ASA/IR-ASA | - AUC_0-t_ (area-under-the-curve) - AUC_0-∞_ (AUC_0-t_ extrapolated to infinity) - C_max_ (maximum plasma concentration) - t_max_ (time of peak drug concentration) - λ_z_ (terminal elimination rate constant) - t_½_ (first-order elimination half-life) - V_D_/F (apparent volume of distribution)* - CL/F (oral clearance)* |
| % inhibition of serum thromboxane B2 (TxB2) levels | - AUC_0-24_ (area-under-the-curve) - I_max_ (maximum % inhibition of TxB2 levels) - t_max_ |
| **Secondary** | - the ratio of least-square-means (LSM) of AUC_0-t_, AUC0-∞, and C_max_ of salicylic acid for PL-ASA and IR-ASA - AUC_0-t_, AUC0-∞, C_max_, t_max_, λz, t_½_, V_D_/F, and CL/F of acetylsalicylic acid for PL-ASA and IR-ASA - the ratio of LSM of AUC_0-t_, AUC_0-∞_, and C_max_ of acetylsalicylic acid for PL-ASA and IR-ASA - the incidence of aspirin responders as assessed by ≥95% inhibition of serum TxB2 levels, and by the incidence of subjects with ≤1500 pg/mg creatinine of urinary 11-dehydro-TxB2 - the level of platelet aggregation in response to arachidonic acid and collagen, determined by an ex vivo assay |

**Statistical Methods**

Analyses of variance (ANOVA) were performed on the natural log-transformed AUC_0-t,_ AUC_0-∞_ and C_max_ of PL-ASA to IR-ASA. The ANOVA MIXED model included aspirin formulation (PL-ASA or IR-ASA) as a fixed factor, with subjects within sequence included as a random effect. LSM was calculated using the exponentiation of the LSM from the analyses of the log-transformed AUC_0-t_, AUC_0-∞_ and C_max_. The 90% CIs for the ratios were derived by exponentiation of the CIs obtained for the difference between group LSM resulting from the analyses on the log-transformed AUC_0-t_, AUC_0-∞_ and C_max_. The ratios of LSM and 90% CIs were expressed as a percentage of PL-ASA to IR-ASA. The 2 aspirin drug products were considered bioequivalent if the 90% CIs of the geometric mean ratios for AUC_0-t,_ AUC_0-∞,_ and C_max_ for PL-ASA and IR-ASA were within the 80% to 125% interval accepted by the FDA to demonstrate bioequivalence.

An assessment of outliers was conducted, taking into consideration the FDA Guidance "Statistical Approaches to Establishing Bioequivalence"^[[1]](#endnote-1)^(14). Testing for outliers was performed for both salicylic acid and acetylsalicylic acid for the log-transformed PK parameters AUC_0-∞_, AUC_0-t_, and C_max_ following both 325-mg and 650-mg doses. Based upon the analyses results, and recognizing that the exclusion of outliers should be reserved for those instances where an identified statistically significant result is obvious, and is consistent across PK parameters (one subject was excluded from the salicylic acid 325-mg dose group and two subjects from the salicylic acid 650-mg dose group). Outlier analyses indicated these same exclusions could be made for acetylsalicylic acid PK parameter calculations. Determination of the PK profiles of PL-ASA and IR-ASA based on salicylic acid and acetylsalicylic acid, and bioequivalence analyses for log-transformed PK parameters AUC_0-t_, AUC_0-∞_ and C_max_ for the ratio of PL-ASA to IR-ASA, were performed in 2 ways (both with and without these outlier exclusions) to illustrate the effect of outliers on the analyses.

The primary endpoints for evaluating the PD of IR-ASA and PL-ASA were the PD parameters AUC_0-24_, I_max_, and t_max_ for the percent inhibition of serum TxB2 levels. PD equivalence of PL-ASA and IR-ASA was determined by the use of a fixed-effects repeated measures ANOVA model, utilizing the SAS GLM procedure for the log-transformed TxB2 parameters AUC_0-24_, I_max_, and t_max_. The model included fixed effects of drug, period, and sequence, with subjects within sequence included as a fixed effect. Other analyses for these TxB2-related endpoints were conducted in a fashion parallel to that described above for the PK endpoints of salicylic acid.

*Two pharmacokinetic parameters were to be determined (apparent volume of distribution [V/F] and apparent clearance [CL/F]), but they were not calculated as neither parameter was necessary for bioequivalence determinations

**Online Table 2. Demographics of the study population**

|  | **325-mg Dose**  (n = 16) | **650-mg Dose**  (n = 16) | **Overall**  (n = 32) |
| --- | --- | --- | --- |
| **Age,** mean ± SD | 36.7 ± 9.9 | 36.9 ± 9.5 | 36.8 ± 9.6 |
| **Sex** |  |  |  |
| Male, n (%) | 6 (37.5) | 6 (37.5) | 12 (37.5) |
| Female, n (%) | 10 (62.5) | 10 (62.5) | 20 (62.5) |
| **Ethnicity** |  |  |  |
| White, n (%) | 8 (50.0) | 10 (62.5) | 18 (56.3) |
| Native Hawaiian/Islander, n (%) | 1 (6.2) | 0 (0) | 1 (3.1) |
| African American, n (%) | 7 (43.8) | 6 (37.5) | 13 (40.6) |
| **Height** (in) | 66.5 ± 4.3 | 68.8 ± 3.7 | 67.7 ± 4.1 |
| **Weight** (pounds) | 167.4 ± 55.7 | 174.3 ± 39.2 | 170.8 ± 47.5 |
| **Subjects Completed Study Per Protocol** | 14 (87.5%) | 16 (100%) | 16 (100%) |
| Unable to confirm baseline platelet  aggregation at Study Visit 3 | 2 (12.5%) | 0 (0%) | 2 (6.2%) |

**Online Table 3. Pharmacokinetics of PL-ASA and IR-ASA (acetylsalicylic acid) in healthy volunteers**

| **Acetylsalicylic acid 325 mg Dose** | | | | | | | |
| --- | --- | --- | --- | --- | --- | --- | --- |
| **Parameter** |  | **PL-ASA** | |  | **IR-ASA** | | |
|  | n | median | range | n | median | range |  |
| AUC_0-t_ (μg×min/mL) | 13 | 205 | 104 - 269 | 13 | 214 | 68 - 287 |  |
| AUC0-infinity (µg ×min/mL) | 9 | 191 | 117 - 267 | 9 | 234 | 142 -292 |  |
| C_max_ (μg /mL) | 13 | 3.0 | 1.5 - 4.4 | 13 | 2.3 | 0.8 - 5.0 |  |
| t_max_ (min) | 13 | 60 | 40 - 180 | 13 | 60 | 20 - 240 |  |
| λ(1/min) | 9 | 0.02 | 0.005 – 0.047 | 9 | 0.02 | 0.011 – 0.042 |  |
| t_½_ (min) | 9 | 34 | 15 - 129 | 9 | 29 | 16 - 64 |  |
| **Acetylsalicylic acid 650 mg Dose** | | | | | | | |
| **Parameter** |  | **PL-ASA** | |  | **IR-ASA** | | |
|  | n | median | range | n | median | range |  |
| AUC_0-t_ (μg×min/mL) | 14 | 378 | 213 – 526 | 14 | 385 | 282 – 697 |  |
| AUC_0-infinity_ (µg ×min/mL) | 11 | 345 | 225 – 508 | 14 | 402 | 289 – 773 |  |
| C_max_ (μg /mL) | 14 | 5 | 1 – 10 | 14 | 5 | 3 – 10 |  |
| t_max_ (min) | 14 | 60 | 25 – 180 | 14 | 40.0 | 25 – 90 |  |
| λ(1/min) | 11 | 0.03 | 0.01 – 0.05 | 14 | 0.02 | 0.002 – 0.055 |  |
| t_½_ (min) | 11 | 23 | 15– 68 | 14 | 28.7 | 13 – 288 |  |

AUC_0-t_ =area-under-the-curve)**,** AUC_0-∞_ =AUC_0-t_ extrapolated to infinity, C_max_ =maximum plasma concentration, IR-ASA= immediate release aspirin, μg=micrograms, mg=milligrams, min=minutes, mL=milliliters, , PL-ASA=pharmaceutical lipid-aspirin complex, t_max_ =time of peak drug concentration, λ_z_ =terminal elimination rate constant, t_½_ =first-order elimination half-life

**Online Table 4. Summary of Inhibition of TxB2 Parameters for PL-ASA and IR-ASA**

| **325 mg Dose** | | | | | | |
| --- | --- | --- | --- | --- | --- | --- |
|  | **PL-ASA**  (n=15) | | | **IR-ASA**  (n=14) | | |
|  | **Mean** | **CV (%)** | **Median (range)** | **Mean** | **CV (%)** | **Median (range)** |
| **AUC_0-t_**  (% inh×min) | 125440.4 | 2.3 | 125138.3  (119439.5 - 130032.0) | 125078.2 | 1.8 | 124463.3  (123002.2 - 131707.5) |
| **I_max_** (%) | 99.9 | 0.2 | 100.0  (99.1 - 100.0) | 100.0 | 0.00 | 100.0  (100.0 - 100.0) |
| **t_max_** (min) | 837.7 | 62.4 | 600.0  (120.0 - 1469.0) | 683.6 | 61.9 | 600.0  (240.0 - 1449.0) |
| **650 mg Dose** | | | | | | |
|  | **PL-ASA**  (n=15) | | | **IR-ASA**  (n=15) | | |
| **AUC_0-t_**  (% inh×min) | 125045.3 | 2.5 | 123800.0  (120141.2 - 132338.2) | 124525.4 | 5.7 | 125100.8  (100288.04 - 131483.01) |
| **I_max_** (%) | 100.0 | 0.00 | 100.0  (100.0 - 100.0) | 100.0 | 0.00 | 100.0  (100.0 - 100.0) |
| **t_max_** (min) | 749.1 | 47.1 | 600.0  (480.0 - 1443.0) | 880.4 | 50.7 | 600.0  (360.0 - 1484.0) |

AUC_0-t_ =area-under-the-curve, CV=coefficient of variation, C_max_ =maximum plasma concentration, IR-ASA= immediate release aspirin, inh=inhibition, mg=milligrams, min=minutes, mL=milliliters, PL-ASA=pharmaceutical lipid-aspirin complex, t_max_ =time of peak drug concentration

**Online Table 5. Summary of Log-Normalized Ratio of PL-ASA to IR-ASA for Statistics Based on Inhibition of TxB2 (325 mg and 650 mg doses)**

|  | **PL-ASA** | **IR-ASA** | **Ratio^*^ (%)** | **90% CI^†^** | **ANOVA**  **p-value**‡ |
| --- | --- | --- | --- | --- | --- |
| **325 mg Dose** | | | | | |
| **AUC0-t** (% inh×min) | 125379.4 | 125241.6 | 100.28 | (98.88, 101.69) | 0.6702 |
| **Imax** (%) | 99.9 | 100.0 | 99.94 | (99.81, 100.07) | 0.4281 |
| **650 mg Dose** | | | | | |
| **AUC0-t** (% inh×min) | 125078.9 | 123921.1 | 100.56 | (97.00, 104.26) | 0.7536 |
| **Imax** (%) | 100.0 | 100.0 | 100.0 | (100.00, 100.00) | --- |

*Ratio = 100 × Geometric Mean (PL-ASA/ Geometric Mean (IR-ASA)

^†^90% Confidence Interval on the Ratio of PL-ASA to IR-ASA

‡p-value for the difference in the treatment estimates; significant difference is defined as p-value <0.05.

AUC_0-t_ =area-under-the-curve, CI=confidence interval, inh=inhibition, IR-ASA= immediate release aspirin, mg=milligrams, min=minutes, PL-ASA=pharmaceutical lipid-aspirin complex, TxB2=thromboxane B2, t_max_ =time of peak drug concentration

**Online Table 6. Incidence of Aspirin Responders as Assessed by % Inhibition of Serum Thromboxane B2 Levels**

| **Criterion for Aspirin Responders**  **(% Inhibition of Serum TxB_2_ Levels)** | **Incidence of Aspirin Responders**  **Number of Responders / Number of Evaluable Subjects (%)** | | | |
| --- | --- | --- | --- | --- |
|  | **PL-ASA** | | **IR-ASA** | |
|  | **325 mg** | **650 mg** | **325 mg** | **650 mg** |
| At least 90% | 13/13 (100.0%) | 14/14 (100.0%) | 13/13 (100.0%) | 14/14 (100.0%) |
| At least 95% | 13/13 (100.0%) | 14/14 (100.0%) | 13/13 (100.0%) | 14/14 (100.0%) |
| At least 99% | 13/13 (100.0%) | 14/14 (100.0%) | 13/13 (100.0%) | 14/14 (100.0%) |

1. IR-ASA= immediate release aspirin, PL-ASA=pharmaceutical lipid-aspirin complex, TxB2=thromboxane B2 [↑](#endnote-ref-1)
